# Catalysts with Cerium in a Membrane Reactor for the Removal of Formaldehyde Pollutant from Water Effluents

**DOI:** 10.3390/molecules21060668

**Published:** 2016-05-24

**Authors:** Mirella Gutiérrez-Arzaluz, Luis Noreña-Franco, Saúl Ángel-Cuevas, Violeta Mugica-Álvarez, Miguel Torres-Rodríguez

**Affiliations:** Area of Applied Chemistry, Universidad Autónoma Metropolitana, Azcapotzalco, México City 02200, Mexico; lnf@correo.azc.uam.mx (L.N.-F.); saul_cuevas00@hotmail.com (S.Á.-C.); vma@correo.azc.uam.mx (V.M.-Á.); trm@correo.azc.uam.mx (M.T.-R.)

**Keywords:** catalytic wet oxidation, formaldehyde, cerium oxide, cobalt oxide

## Abstract

We report the synthesis of cerium oxide, cobalt oxide, mixed cerium, and cobalt oxides and a Ce–Co/Al_2_O_3_ membrane, which are employed as catalysts for the catalytic wet oxidation (CWO) reaction process and the removal of formaldehyde from industrial effluents. Formaldehyde is present in numerous waste streams from the chemical industry in a concentration low enough to make its recovery not economically justified but high enough to create an environmental hazard. Common biological degradation methods do not work for formaldehyde, a highly toxic but refractory, low biodegradability substance. The CWO reaction is a recent, promising alternative that also permits much lower temperature and pressure conditions than other oxidation processes, resulting in economic benefits. The CWO reaction employing Ce- and Co-containing catalysts was carried out inside a slurry batch reactor and a membrane reactor. Experimental results are reported. Next, a mixed Ce–Co oxide film was supported on an γ-alumina membrane used in a catalytic membrane reactor to compare formaldehyde removal between both types of systems. Catalytic materials with cerium and with a relatively large amount of cerium favored the transformation of formaldehyde. Cerium was present as cerianite in the catalytic materials, as indicated by X-ray diffraction patterns.

## 1. Introduction

The catalytic wet oxidation processes fall within the three-phase reaction categories, which are somewhat difficult to operate and are therefore not in a state of technological readiness for commercial application. Thus, it is widely recognized that the technology of catalytic wet air oxidation (CWAO) needs further development to become a commercial option, economically viable, environmentally responsible, and a way to remove the organic waste generated and increase the possibility of managing emerging problems in the treatment of toxic wastewater.

In wet air oxidation (WAO) processes, organic pollutants dissolved in water are degraded, either partially through an oxidizing agent in biodegradable products or mineralized into harmless inorganic compounds, such as CO_2_, H_2_O, and inorganic salts, which remain in the liquid phase [1].

Compared to the WAO process, the presence of a catalyst in CWAO has lower energy requirements [2]; in addition, it has the advantage of reaching a greater oxidation conversion. Consequently, less severe conditions can be used to reduce the chemical oxygen demand because in this process, organic compounds are oxidized to inorganic usable by-products, such as CO_2_ and H_2_O.

Wet air oxidation [3] represents an alternative technology to treat water streams with a low concentration of toxic organic compounds. However, the absence of a catalyst implies that high temperatures and oxygen pressures are required [4]. However, the use of catalysts could diminish temperature and air pressure requirements, resulting in a more efficient process of abating organic agents. There is considerable potential for this catalytic wet air oxidation process to ultimately destroy organic pollutants in industrial effluents [1]. 

Nevertheless, despite this great effort in catalyst formulations, several problems can remain in this process [1], for example, obtaining efficient contact among gas, liquid, and solid phases in a process that is easily limited by the transfer of the gaseous reactant. Relatively few innovations have been published concerning the CWAO of organic agents in trickle bed reactors [5,6,7] to improve these gas/liquid/solid contact performances. Moreover, in reactors with high liquid-to-catalyst volumetric ratios (such as slurry and bubble column fixed-bed reactors), carbonaceous deposition on the surface of catalyst particles is enhanced because oxidative coupling reactions are favored in the bulk liquid phase [8].

One method of improving the gas/liquid/solid contact could be to use contactor type catalytic membrane reactors (CMR) [9,10,11] as interfacial contactors with an organic compound solution and air (or oxygen) separately fed from both sides of the catalytic membrane. The gas overpressure can shift the gas-liquid interface location into the membrane wall, which is closer to the catalytic zone, thus realizing several advantages: the oxygen concentration in contact with the catalyst layer is maximized, the desorption of pollutants into the gaseous phase is favored, the catalyst exposure to leaching is reduced, substantial flexibility for operative conditions is available and scale-up issues are facilitated. Conversely, the proper location of the gas-liquid interphase must be ensured to take advantage of the previously cited benefits.

Formaldehyde (HCHO) is an important environmental pollutant because of its adverse health effects [5] and has been chosen as a model pollutant for the wet catalytic oxidation reaction. Among the several metal oxides with catalytic properties, we selected CeO_2_ because of its high oxygen storage/transport capacity (OSC) and particular redox properties [12]. The incorporation of other oxides, such as cobalt oxide, into the ceria lattice enhances redox properties, providing high thermal stability. The Ce–Co mixed oxides are more resistant to poisoning phenomena and are generally less active than supported precious metals for oxidizing VOC streams [13]. Considerable research efforts have focused on catalyst preparation, thus obtaining Ce–Co mixed oxides with a large surface area. Among the synthesis methods, the co-precipitation procedure from the corresponding salts has found a recurrent application attributable to the high oxygen storage capacity achieved by the final material [14,15]. The addition of an appropriate amount of CeO_2_ could increase the surface area of Co_3_O_4_ and improve the redox ability [16] of Co^2+^/Co^3+^. This work was intended to evaluate materials’ catalytic properties based on Ce and Co oxides in two arrangements: (1) in bulk; and (2) supported on a mesoporous alumina membrane. Conventional bulk catalysts have pores with a single entrance, whereas a porous catalytic membranes have interconnected pores with open ends towards two faces. The catalytic evaluation was performed both in a slurry-type batch reactor (the bulk catalyst) and in a membrane reactor (the supported catalyst). 

## 2. Results and Discussion

### 2.1. Bulk Catalyst Characterization

**Figure 1:**
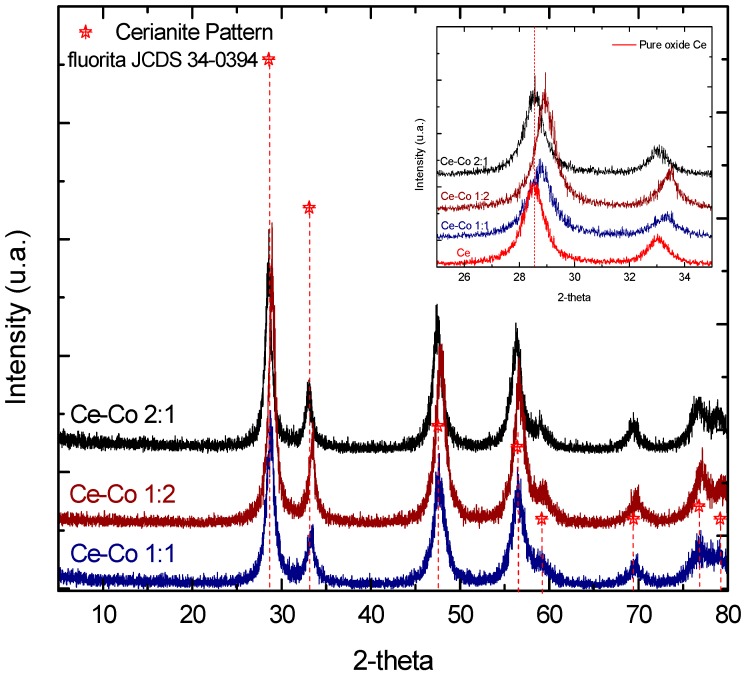
XRD patterns of the mixed Ce–Co oxides: 1:1, 1:2, and 2:1.

The X-ray diffraction patterns of the mixed oxides Ce–Co with 1:1, 2:1, and 1:2 molar ratios are presented in Figure 1, along with all of the samples exhibiting the cerianite structure (CeO_2_) with characteristic peaks at the (111), (200), (220), (311), (222), and (400) planes [17,18]; these patterns do not exhibit corresponding peaks for the cobalt oxide [19,20].

Apparently, the effect of cobalt oxide is to reduce the intensity of the peaks, probably either because of the comparatively small size of the cobalt oxide particles or because some of the cobalt oxide is incorporated into the cerianite framework [17,18]. The diffraction pattern was not affected by the relative molar ratio, and the same structure was observed for all three Ce–Co oxides molar ratios (1:1, 1:2, and 2:1).

Employing Scherrer´s equation, we discovered that the particle size changed after the incorporation of the cobalt oxide into the cerium oxide. The pure cerium oxide had a 372 Å particle size, whereas the particle size of the 1:1 Ce–Co mixed oxide was 365.2 Å. For the 2:1 Ce–Co mixed oxide, the particle size has increased slightly to 378.5 Å, and for the 1:2 Ce–Co mixed oxide, the particle size increased to 850 Å. Furthermore, taking the (111) diffraction peak at 28.5° of the XRD pattern of the JCDS 34-0394 fluorite as reference and considering the XRD pattern of pure cerium oxide, we observed a slight displacement of the peak position (at 28.8°) that corresponded to a parameter lattice of 5.37 Å for the 1:1 Ce–Co mixed oxide; this displacement was further increased (29°) with a parameter lattice of 5.33 Å for the 1:2 Ce–Co sample. The 2:1 Ce–Co sample did not exhibit a diffraction peak shift, had a larger crystal size, and had a parameter lattice of 5.41 Å, which is the same as that of pure oxide.

**Figure 2:**
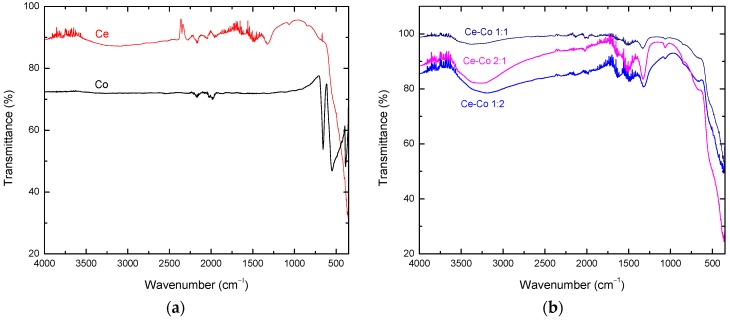
FTIR spectra of the (**a**) pure oxides: Ce and Co; (**b**) mixed oxides: Ce–Co 1:1, Ce–Co 1:2, Ce–Co 2:1.

Figure 2a shows the FTIR spectra of the cerium and cobalt pure oxides. We observed the characteristic absorption band of cobalt at approximately 500 cm^−1^, which is associated with the vibrational modes of the Co_3_O_4_ spinel phase [21]. Figure 2b shows the FTIR spectra of the Ce and Co mixed oxides, which exhibit small absorption bands at approximately 1316 and 1492 cm^−1^ that correspond to inorganic carboxylates (Ce–CoO) [20,22]. We should note that in the FTIR spectra of the three different molar ratios of the Ce and Co mixed oxides, there was no evidence of the cobalt spinel phase.

**Figure 3:**
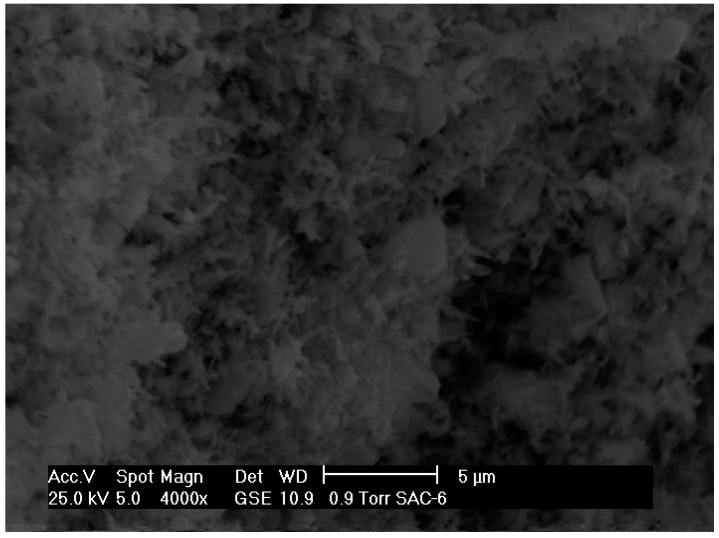
SEM micrograph of mixed oxide: Ce–Co 1:1.

**Figure 4:**
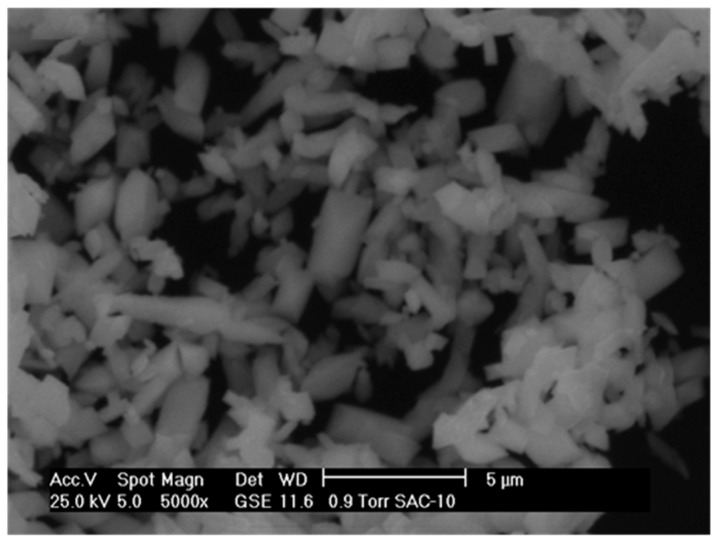
SEM micrograph of mixed oxide: Ce–Co 2:1.

**Figure 5:**
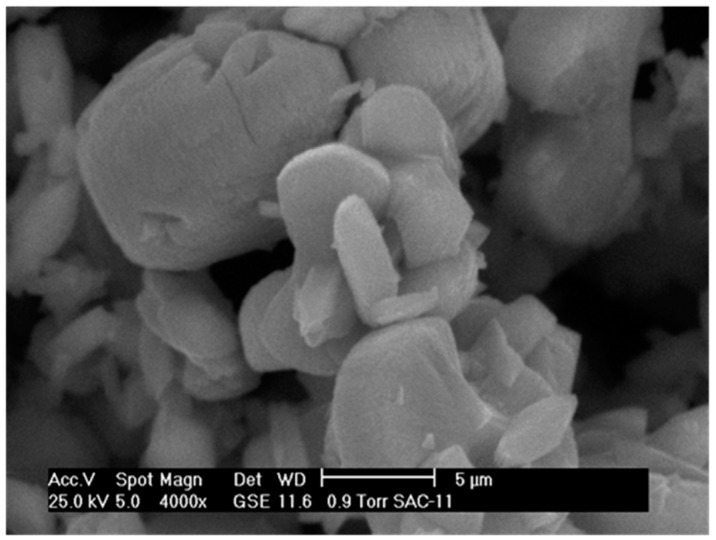
SEM micrograph of mixed oxide: Ce–Co 1:2.

Figure 3, Figure 4 and Figure 5 show the micrographs of the cerium and cobalt mixed oxides, and we can observe that by varying the Ce/Co molar ratio (1:1, 1:2, and 2:1, respectively), larger and more irregularly shaped particles were obtained. Compared to the 1:1 Ce–Co sample (Figure 3), the sample with the high cerium content had larger and sharper particles (Figure 4). The sample with the high cobalt content (Figure 5) had a sort of sponge morphology formed by an inter-crossing laminate. Therefore, we can infer that increasing the Co content promotes both a change in morphology and larger particle clusters. SEM images correlate to XRD results, showing that higher Co content increases the particle size.

### 2.2. Catalytic Membrane Characterization

**Figure 6:**
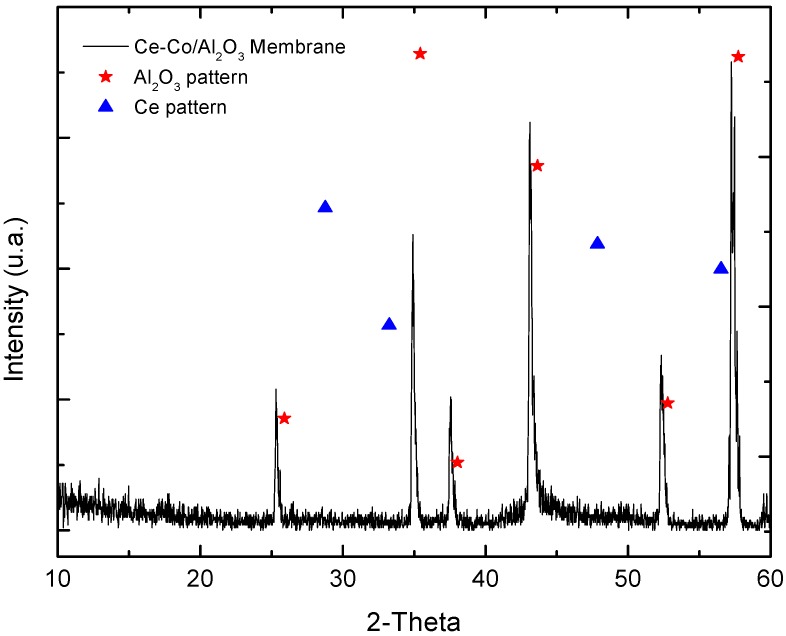
XRD pattern of the Ce–Co/Al_2_O_3_ membrane.

We will describe the XRD results of the Ce–Co/Al_2_O_3_ membrane both as a reference and for comparison with the membrane that was used for the HCOH CWO reaction. Figure 6 shows the XRD pattern, presenting the Al_2_O_3_ characteristic peaks of the corundum phase with a rhombohedral crystal cell [23]. The alumina peaks were slightly shifted towards smaller diffraction angles. We could not see either the cobalt oxide or the cerium oxide XRD signals, indicating that the mixed oxide film is relatively thin and that when using conventional 2θ scanning methods for supported film, very thin film generally produces a weak signal from the film and an intense signal from the substrate, in this case alpha alumina [24,25]. In addition, the diffraction peaks of alpha alumina support were slightly displaced towards lower angles relative to the pattern; we associate this displacement with the stiffness and geometry of the support.

**Figure 7:**
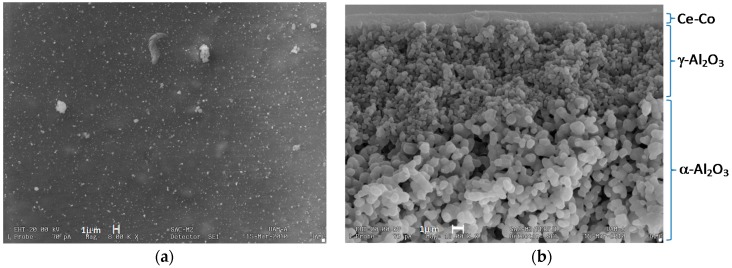
SEM micrographs of Ce–Co/Al_2_O_3_ membrane, (**a**) surface morphology, and (**b**) cross-section.

Figure 7 shows the Ce–Co/Al_2_O_3_ membrane micrographs. We present a representative section of the inner side surface (Figure 7a), noticing a relatively uniform surface with no fractures or pinhole. Figure 7b shows the cross-section image with three of the four layers of the alumina support and the top Co-Ce film, featuring a considerably uniform thickness of approximately 1 µm.

**Figure 8:**
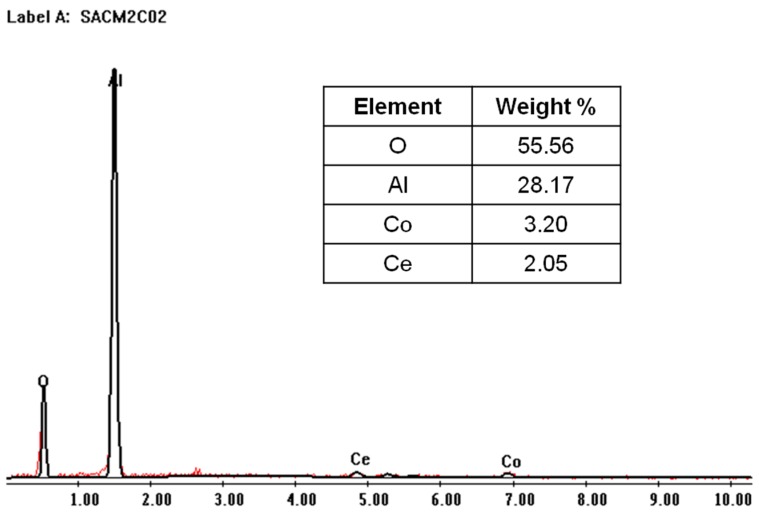
SEM/EDS spectra of the Ce–Co/Al_2_O_3_ membrane.

Figure 8 describes the elemental analysis obtained with the SEM-EDS technique, verifying the presence of cobalt and cerium in the film with the alumina support. We also notice a higher percentage cobalt content with respect to cerium content. The elemental analysis indicates a high level of oxygen and aluminium belonging to the alumina support. Other elements are impurities.

Considering the SEM results, the Co and Ce mixed oxides impregnation method was effective for the growth of a uniform film of homogeneous thickness. For this method, sequential 2 M precursor solutions for each oxide were used, beginning with cobalt and followed by cerium, and a precipitation step was included for each oxide. A 15-cm-long catalytic membrane for the HCOH CWO reaction was prepared in this manner.

### 2.3. Catalytic Wet Oxidation Reaction

#### 2.3.1. Commercial Autoclave Slurry Batch-Type Reactor

**Figure 9:**
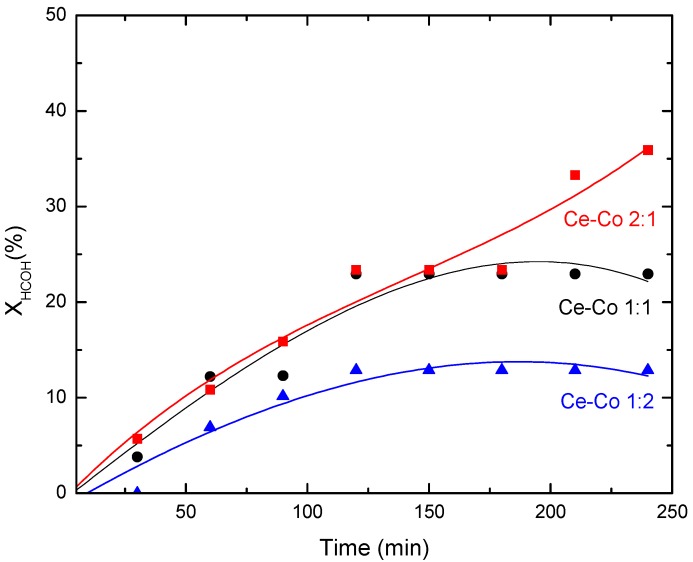
CWO of HCOH with mixed oxides: Ce–Co 1:1, Ce–Co 1:2, Ce–Co 2:1.

Figure 9 shows the conversion time compared to the reaction time of the HCOH CWO reaction that employed three different Ce–Co powder mixed oxides with 1:1, 2:1, and 1:2 Ce–Co molar ratios. Conversion maintains an ascending linear trend from the first minute of the reaction through 90 min; afterwards, for the 1:1 and 1:2 Ce–Co samples, conversion becomes relatively constant, whereas the 2:1 Ce–Co sample continues to show an increase in conversion, achieving an approximately 35% HCOH conversion. Overall, the conversion obtained was smaller than 40%, but we should consider the relatively high initial formaldehyde content (1000 ppm). The oxidation of HCOH leads to various secondary products, such as oxalic acid, formic acid, and acetic acid, depending on the degree of oxidation; moreover, we should note that no secondary products were detected by gas chromatography of the liquid stream. Other authors have reported the production of low molecular weight carboxylic acids and, more importantly, formic acid, a highly refractive compound, when carrying out the HCOH CWO reaction [26,27]. In previous work [28], which was also during formaldehyde removal, the presence of carboxylic acids as secondary reaction products using a Pt/Al_2_O_3_ powder catalyst was observed. 

According to the HCOH conversion results, the 2:1 Ce–Co sample exhibited higher activity than the 1:2 Ce–Co sample, indicating that a higher cerium content promotes HCOH oxidation because of the high capacity of Ce for oxygen storage, as reported in the literature [29]. We measured a 5.75 × 10^−3^ mol·(L·min)^−1^ initial reaction rate for the 1:1 Ce–Co catalyst and a 6.08 × 10^−3^ mol·(L·min)^−1^ initial reaction speed for the 2:1 Ce–Co catalyst, whereas the 1:2 Ce–Co catalyst had an initial reaction speed of 4.00 × 10^−3^ mol·(L·min)^−1^, which is approximately twice as low as the sample with high cerium content. The catalyst with the higher Ce content, the 2:1 Ce–Co exhibiting the higher catalytic activity, had a small particle size.

A similar HCOH conversion of 38.1%, after a 3 h reaction at 200 °C and 15 bar of oxygen pressure with a prolonged induction step, was reported by Silva *et al.* [5,26] for the oxidation reaction with no catalyst (WO). Our conversion rate was likely affected by the high HCOH concentration (1000 ppm), considering that Tang *et al.* [30] report a reduction in conversion when the HCOH initial concentration increased from 100 to 580 ppm.

#### 2.3.2. Catalytic Membrane Reactor, Contactor type

**Figure 10:**
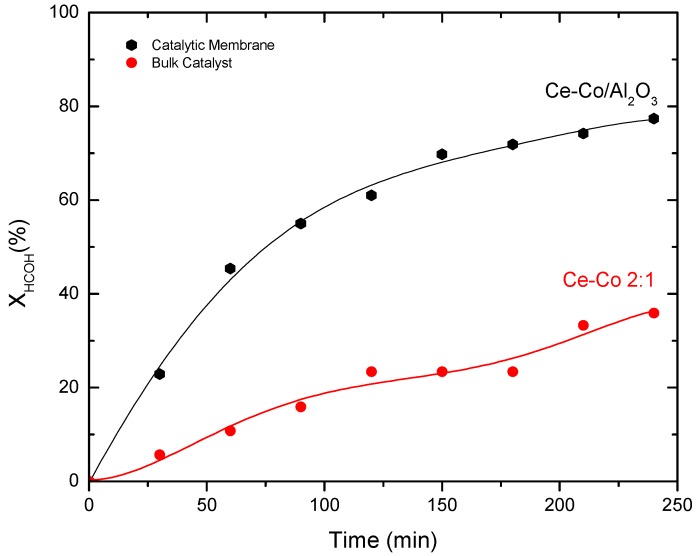
CWO of HCOH, comparison of catalytic membrane (Ce–Co/Al_2_O_3_) and bulk catalyst (Ce–Co 2:1).

Figure 10 shows the catalytic evaluation of the Ce–Co mixed oxide/alumina membrane reactor after 240 min reaction, achieving a 79% total conversion. At the beginning of the reaction, conversion followed an increasing trend, with an almost linear behavior for the first 60 min. The measured initial reaction speed was 16.5 × 10^−3^ mol·(L·min)^−1^. Subsequently, conversion increased at a slower pace. As with powder catalysts, we found no secondary products in the liquid phase (as determined by gas chromatography), and the reaction products were those corresponding to HCOH mineralization.

Figure 10 shows the conversion rate for the two catalytic materials: the bulk Ce–Co mixed oxide (employing a slurry batch reactor) and the Ce–Co mixed oxide supported on mesoporous alumina (as part of a membrane reactor). We attempted to maintain the same reaction conditions: 100 °C temperature, 250 mL reaction volume, 1000 ppm initial HCOH concentration, and 5 bar oxygen pressure. However, we used a continuous gas phase feed of 40 mL·min^−1^ O_2_ for the membrane reactor but a discontinuous one for the slurry batch reactor; the catalyst mass to the reaction volume ratio was also not the same, at 1 g·L^−1^ for the conventional slurry batch reactor and 0.10 g·L^−1^ for the membrane reactor, similar to previous work [11]. The high conversion obtained with the catalytic membrane reactor can be explained by the efficient contact between the liquid phase and the gas phase, specifically at the membrane’s active surface, via a pressure differential between the two phases. The contact between the liquid and gas phases occurs at a 1 µm-thick section, enabling very small mass transference limitations. Such mass transfer limitations are considerable in a slurry type reactor [1].

The mineralization of HCOH was corroborated for the two types of reactors after the CWO reaction with the FTIR analysis of the CaCO_3_ precipitate. The band’s characteristic of calcite transmittance was identified at 713 and 877 cm^−1^ as vibrations of C-O in plane and out of plane, respectively [31,32]; the band at approximately 1400 cm^−1^ corresponded to the bending C=O [33].

Furthermore, the measured pH of the formaldehyde solution before the CWO reaction was 6.3, and after reaction with the mixed oxide catalyst powder the first two reactions (Ce–Co 1: 1 and Ce–Co 1: 2), the pH slightly decreased (to approximately 5.4). However, reactions with Ce–Co 2:1 had a pH of 5.0, and those with the membrane showed an important decrease to a pH of 4.5. The decreased pH in the membrane case probably occurred because of the dissolution of the CO_2_ obtained by HCOH mineralization, which was dissolved in the solution, resulting in a more acidic pH.

## 3. Discussion

Even if the amount of catalyst employed in the membrane reactor was approximately 10 times lower than that of the slurry batch reactor, the reaction speed (and thus the reaction kinetics) obtained with the membrane reactor was approximately three times faster (with the 2:1 Ce–Co catalyst). The conversion curve trend for the membrane reactor shows that the contactor-type configuration exhibits a higher conversion compared to the conversion slurry batch reactor throughout the reaction time; the higher slope registered for CMR suggests a greater local concentration of active oxygen in the membrane film, showing similar reaction times that produce an increase in the highest reaction rate of oxidation by increasing local oxygen concentration. As mentioned in Section 2.3.1, no side products were identified for CMR compared to secondary products in the batch reactor, suggesting higher selectivity for CMR towards mineralization products. 

The results can be related to several factors, including a higher local oxygen concentration inside the pores and a better distribution of the active sites. We should also consider the liquid-solid volumetric ratio, which is also related to the formation of carbonous deposits [1,34]. Slurry-type batch reactors have a high liquid-catalyst volumetric ratio that favors the formation of polymer species in the liquid phase through either addition reactions or condensation reactions of intermediate species. With small liquid-catalyst volumetric ratios (such as in trickle-bed reactors and membrane reactors), the liquid phase is specifically in contact with the solid active layer and therefore propagation reactions in the liquid phase are suppressed, producing an almost quantitative transformation of formaldehyde into CO_2_; fact that is corroborated by the formation of calcium carbonate, formed by the capture of CO_2_ and pH more acidic.

The advantages of the membrane reactor arise out of the gas-liquid contact in the solid catalytic thin mesoporous layer formed by a nanometre-size open-pore system, whereas the bulk solid catalyst is formed mainly by a closed-pore system that is much more prone to blocking by deposits. In summary, the highest conversion of formaldehyde (Figure 10) shows that the membrane reactor-type contactor arises out of the gas-liquid contact at the solid catalytic thin mesoporous layer formed by a nanometre-size open-pore system. The membrane reactor has the additional advantage of not needing the separation of the reaction products from the catalyst after the reaction, as required by conventional batch reactors.

## 4. Materials and Methods 

The preparation of pure Ce and Co oxides and Ce–Co mixed oxide-based catalysts was carried out by the co-precipitation method through controlled additions of the precursor solution containing an excess basic agent (CO(NH_2_)_2_) followed by a calcination stage at 350 °C, as reported in previous works [35]. Briefly, to obtain pure Ce and Co oxides and Ce–Co mixed oxides, 2 M solutions of Co(NO_3_)_2_ and Ce(NO_3_)_3_ were employed as precursors, as provided by Sigma-Aldrich Química, S.L. (Toluca, Mexico). Urea was used as precipitating agent. Ce–Co mixed oxides were prepared with three different molar ratios: 1:1, 2:1, and 1:2. After precipitation, all of the catalytic materials were washed with deionized water, dried for 16 h at ambient temperature and calcined at 350 °C for 2 h under an N_2_ atmosphere at a heating rate of 3 °C·min^−1^. According to Chen and Isa [36], urea decomposes into volatile by-products at 190 °C.

We will also describe the preparation steps of the inorganic Ce–Co/γ-Al_2_O_3_ membrane. Ce–Co films were prepared on the inner surface of an alumina tubular support (Pall Exekia, Bazet, France) with 7 and 10 mm i.d. and o.d., respectively, with an average pore size of 5 nm and a thickness of 10 μm on the top layer (γ-Al_2_O_3_); the remainder of the support is composed of alpha alumina. The length of the permeation section was 120 mm, and both ends of the tubular support were coated with a glass sealant. The alumina supports were first cleaned ultrasonically in acetone and rinsed in deionized water. Prior to film growth, the γ-alumina support was heated at 110 °C for 24 h and covered with Teflon tape (the outer side) to promote film growth only on the inner side. The commercial support was impregnated with a 2 M Co(NO)_3_ 6H_2_O precursor dissolution for 10 min at 95 °C, then with a 4 M urea solution for 1 h at 95 °C, then with a 2 M Ce(NO)_3_ 6H_2_O precursor solution for 10 min at 95 °C and finally with a 4 M urea solution for 1 h at 95 °C. Next, the tube was washed using deionized water and dried for 12 h at 120°C; further calcinations were performed at 350 °C for 3 h in a laboratory-air atmosphere.

The samples were characterized using various techniques such as powder X-ray diffraction (XRD), on a Philips X-pert diffractometer (Nottingham, UK) using a Cu K_α_ radiation source with a goniometer of a 2-theta angle range between 4° and 80° operating at 40 kV and 25 mA. Elemental analysis and surface topographic features were observed through scanning electron microscopy (SEM/EDS) (FEI Company, Eindhoven, The Netherlands) employing a Philips XL30 ESEM microscope allowing both high vacuum and ambient operating conditions and a LEO 440 microscope with secondary electrons and EDS detectors for Fourier-transform infrared spectroscopy (FTIR) (Varian Inc., Palo Alto, CA, USA) with a Varian Excalibur 3600 instrument having a 4000 to 400 cm^−1^ wavenumber range for which the samples were finely ground with an agate mortar. 

The performance of the catalytic systems was studied during the catalytic wet formaldehyde oxidation (CWO) reaction at ppm concentrations. The trials were conducted inside a conventional stainless steel “batch” reactor (commercial autoclave Parr Reactor, Parr Instrument Company, Moline, IL, USA) for the liquid phase, using pure oxygen (99.6% purity, Praxair México, Mexico City, Mexico) as an oxidizing agent at a 100 °C reaction temperature. The initial formaldehyde concentration was 1000 ppm. The ratio of catalyst mass to reaction volume was 1 g·L^−1^; testing was conducted at oxidizing agent pressure, namely, 5 bar. The catalytic HCHO oxidation test was studied for 4 h. The reaction progress was monitored by sampling the liquid phase at different times and analyzed by gas chromatography using an HP 5890 series II brand Plus chromatograph with a flame ionization detector (FID) and GC ChemStation software^©^ (Agilent Technologies, Inc., Santa Clara, CA, USA), along with an HP brand PLOT Q capillary column, 30 m long and 0.32 mm in diameter, suitable for separating organic acids and formaldehyde of low molecular weight. 

The CWO reaction with a membrane reactor has been described in our previous work [11]. The membrane reactor was composed of a stainless steel cylinder jacket containing the catalytic membrane. This setup was divided into two chambers; the outer one was used to feed the oxygen and the inner chamber was used to feed the liquid phase with formaldehyde. The reactor configuration used is known as a contactor. The typical conditions for catalytic tests were as follows: 5 bar oxygen pressure and a 0.5 bar differential pressure between the gas and liquid phases. The membrane reactor results presented here have been obtained by working discontinuously during the liquid phase fed to the internal side in which the catalytic layer is located (250 mL recirculated at 20 mL·min^−1^ by a HPLC pump) and continuously for the gas phase (40 mL O_2_·min^−1^). The gas phase was bubbled into a Ca(OH)_2_ 0.5 M solution throughout the reaction time; the qualitative formation of CO_2_ was determined at the end time, and the formation of a precipitate was found. This precipitate was characterized by FTIR with an accessory of ATR (attenuated total reflectance).

## 5. Conclusions

### 5.1. Catalysts

According to the XRD results, the pure cerium oxide and the mixed cobalt-cerium oxides exhibited the characteristic cerianite pattern, with a FCC unit cell. The pure cobalt oxide exhibited a structure corresponding to the spinel phase.

A film of cobalt-cerium mixed oxides was deposited onto a γ-Al_2_O_3_ commercial support. According to SEM images, we attained a uniform, homogeneous film which was crack-free and had approximately 1 µm thickness. EDS elemental analysis confirmed the cobalt and cerium content on the Al_2_O_3_ support, without cluster formation.

### 5.2. Formaldehyde CWO

Regarding the cobalt and cerium oxide bulk catalysts, the highest conversion for the HCOH CWO reaction was obtained with the 2:1 Ce–Co material. In general terms, a relatively high cerium content enhances the reaction conversion. No secondary products were detected in the liquid phase and therefore, all conversion was oriented towards the mineralization products (CO_2_ and H_2_O). 

The highest conversion (of more than 70%) for the CWO reaction of HCOH was obtained with a catalytic membrane of the Co-Ce oxides deposited on the alumina support after a 240 min reaction at 100 °C and 5 bar O_2_. Therefore, the membrane reactor was more efficient than the conventional batch reactor, where the catalyst is in suspension form.

We found that for the membrane reactor-type contactor in which the liquid-catalyst volume ratio is low, propagation reactions or secondary reactions in the liquid phase are eliminated, which results in a nearly quantitative conversion of formaldehyde to CO_2_. 

Coupling the catalytic wet oxidation with the membrane reactor is a promising alternative for the efficient removal of formaldehyde pollutant from industrial residual water, such as in “tailor-made” procedures.

## Abbreviations

The following abbreviations are used in this manuscript:

CWO	Catalytic Wet Oxidation
CWAO	Catalytic Wet Air Oxidation
WAO	Wet Air Oxidation
CMR	Catalytic Membrane Reactors
OSC	Oxygen Storage/Transport Capacity
XRD	X-ray Diffraction
SEM/EDS	Scanning Electron Microscopy/Energy Dispersive Spectroscopy
FTIR	Fourier-Transform Infrared Spectroscopy
GC	Gas Chromatography
FID	Flame Ionization Detector

## References

[1] Levec J., Pintar A. (2007). Catalytic wet-air oxidation processes: A review. Catal. Today.

[2] Busca G., Berardinelli S., Resini C., Arrighi L. (2008). Technologies for the removal of phenol from fluid streams: A short review of recent developments. J. Hazard. Mater..

[3] Zimmerman F.J. (1954). Waste Disposal. U.S. Patent:.

[4] Bhargava S.K., Tardio J., Prasad J., Folger K., Akolekar D.B., Grocott S.C. (2006). Wet Oxidation and Catalytic Wet Oxidation. Ind. Eng. Chem. Res..

[5] Silva A.M.T., Castelo-Branco I.M., Quinta-Ferreira R.M., Levec J. (2003). Catalytic studies in wet oxidation of effluents from formaldehyde industry. Chem. Eng. Sci..

[6] Yang S., Zhu W., Wang J., Chen Z. (2008). Catalytic wet air oxidation of phenol over CeO_2_-TiO_2_ catalyst in the batch reactor and the packed-bed reactor. J. Hazard. Mater..

[7] Pintar A., Batista J., Tišler T. (2008). Catalytic wet-air oxidation of aqueous solutions of formic acid, acetic acid and phenol in a continuous-flow trickle-bed reactor over Ru/TiO_2_ catalysts. Appl. Catal. B Environ..

[8] Pintar A., Levec J. (1994). Catalytic Liquid-Phase Oxidation of Phenol Aqueous Solutions. A Kinetic Investigation. Ind. Eng. Chem. Res..

[9] Miachon S., Perez V., Crehan G., Torp E., Ræder H., Bredesen R., Dalmon J.A. (2003). Comparison of a contactor catalytic membrane reactor with a conventional reactor: Example of wet air oxidation. Catal. Today.

[10] Berčič G., Pintar A., Levec J. (2005). Positioning of the reaction zone for gas–liquid reactions in catalytic membrane reactor by coupling results of mass transport and chemical reaction study. Catal. Today.

[11] Gutiérrez M., Pina M.P., Torres M., Cauqui M.A., Herguido J. (2010). Catalytic wet oxidation of phenol using membrane reactors: A comparative study with slurry-type reactors. Catal. Today.

[12] Trovarelli A., Boaro M., Rocchini E., de Leitenburg C., Dolcetti G. (2001). Some recent developments in the characterization of ceria-based catalysts. J. Alloys Compd..

[13] Chen X., Carabineiro S.A.C., Bastos S.S.T., Tavares P.B., Orfao J.J.M., Pereira M.F.R., Figueiredo J.L. (2014). Catalytic oxidation of ethyl acetate on cerium-containing mixed oxides. Appl. Catal. A Gen..

[14] Xue L., Zhang C., He H., Teraoka Y. (2007). Catalytic decomposition of N_2_O over CeO_2_ promoted Co_3_O_4_ spinel catalyst. Appl. Catal. B Environ..

[15] Hammes M., Stöwe K., Maier W.F. (2012). Cobalt based emission control catalysts with high resistance towards halide poisoning. Appl. Catal. B Environ..

[16] Matheswaran M., Balaji S., Chung S.J., Moon I.S. (2007). Studies on cerium oxidation in catalytic ozonation process: A novel approach for organic mineralization. Catal. Commun..

[17] Faria P.C.C., Monteiro D.C.M., Órfão J.J.M., Pereira M.F.R. (2009). Cerium, manganese and cobalt oxides as catalysts for the ozonation of selected organic compounds. Chemosphere.

[18] Hung I.M., Wang H.P., Lai W.H., Fung K.Z., Hon M.H. (2004). Preparation of mesoporous cerium oxide templated by tri-block copolymer for solid oxide fuel cell. Electrochim. Acta.

[19] Toro A.R., Quispe A., León I. (2005). Preparación y caracterización de electrodos de espinela de cobalto dopados con niquel. REVCIUNI.

[20] Ratnasamy P., Srinivas D., Satyanarayana C.V.V., Manikandan P., Senthil Kumaran R.S., Sachin M., Shetti V.N. (2004). Influence of the support on the preferential oxidation of CO in hydrogen-rich steam reformates over the CuO–CeO_2_–ZrO_2_ system. J. Catal..

[21] Garrido Pedrosa A.M., Souza M.J.B., Melo D.M.A., Araujo A.S., Zinner L.B., Fernandes J.D.G., Martinelli A.E. (2003). Systems involving cobalt and cerium oxides: Characterization and catalytic behavior in the C_6_–C_7_
*n*-alkanes combustion. Solid State Sci..

[22] Holmgren A., Andersson B., Duprez D. (1999). Interactions of CO with Pt/ceria catalysts. Appl. Catal. B Environ..

[23] Feret F.R., Roy D., Boulanger C. (2000). Determination of alpha and beta alumina in ceramic alumina by X-ray diffraction. Spectrochim. Acta.

[24] Tanner B.K., Hase T.P.A., Lafford T.A., Goorsky M.S. (2004). Grazing incidence in-plane X-ray diffraction in the laboratory. JCPDS Adv. X-ray Anal..

[25] Bouroushian M., Kosanovic T. (2012). Characterization of Thin Films by Low Incidence X-ray Diffraction. Cryst. Struct. Theory Appl..

[26] Silva A.M.T., Quinta-Ferreira R.M., Levec J. (2003). Catalytic and Noncatalytic Wet Oxidation of Formaldehyde. A Novel Kinetic Model. Ind. Eng. Chem. Res..

[27] Yang X., Shen Y., Yuan Z., Zhu H. (2005). Ferric ions doped 5A molecular sieves for the oxidation of HCHO with low concentration in the air at moderate temperatures. J. Mol. Catal. A Chem..

[28] Gutiérrez-Arzaluz M., Torres-Rodríguez M., Mugica-Álvarez V., Aguilar-Pliego J., Romero-Romo M. (2016). Wet Oxidation of Formaldehyde with Heterogeneous Catalytic Materials. Int. J. Environ. Sci. Dev..

[29] Li K., Wang H., Wei Y., Liu M. (2008). Catalytic performance of cerium iron complex oxides for partial oxidation of methane to synthesis gas. J. Rare Earths.

[30] Tang X., Chen J., Huang X., Xu Y., Shen W. (2008). Pt/MnO_x_–CeO_2_ catalysts for the complete oxidation of formaldehyde at ambient temperature. Appl. Catal. B Environ..

[31] Islam K., Zuki B., Mustapha M., Mohd Z., Norshazlirah S., Eaqub A. (2011). Characterisation of calcium carbonate and its polymorphs from cockle Shells. Powder Technol..

[32] Rodriguez J., Shaw S., Benning L. (2011). The kinetics and mechanisms of amorphous calcium carbonate (ACC) crystallization to calcite, via vaterite. Nanoscale.

[33] Menahem T., Yitzhak M. (2008). Controlled crystallization of calcium carbonate superstructures in macroemulsions. J. Cryst. Growth.

[34] Pintar A., Levec J. (1992). Catalytic oxidation of organics in aqueous solutions: I. Kinetics of phenol oxidation. J. Catal..

[35] Ángel-Cuevas S., Gutiérrez-Arzaluz M., Aguilar-Pliego J., Mugica-Alvarez V., Noreña-Franco L., Torres-Rodríguez M. (2011). Remoción de formaldehido, de efluentes acuosos mediante oxidación húmeda catalítica. Av. Cienc. Ing..

[36] Chen J.P., Isa K. (1998). Thermal Decomposition of Urea and Urea Derivatives by Simultaneous TG/(DTA)/MS. J. Mass Spectrom. Soc. Jpn..

